# Integrative transcriptomic and proteomic analysis reveals the regulatory mechanisms underlying oilseed rape resistance to Leptosphaeria biglobosa

**DOI:** 10.3389/fpls.2026.1877778

**Published:** 2026-07-15

**Authors:** Yongyi Xia, Haiyan Huangfu, Mengjiao Yan, Ruixuan Zhao, Xinlei Wang, Lili Zhao, Hongli Huo, Chen Guo, Yu Zhou, Ruigang Wang, Ziqin Li

**Affiliations:** 1 Key Laboratory of Plants Adversity Adaptation and Genetic Improvement in Cold and Arid Regions of Inner Mongolia, Inner Mongolia Agricultural University, Hohhot, China; 2 Plant Protection Institute, Inner Mongolia Academy of Agricultural & Animal Husbandry Sciences, Hohhot, China

**Keywords:** *Brassica napus*, *Leptosphaeria biglobosa*, MAPK signaling, proteomics, RNA-seq, transcriptomics

## Abstract

Blackleg disease caused by Leptosphaeria species is a major constraint to rapeseed (*Brassica napus*) production worldwide. Although resistance to *L. maculans* is well studied, molecular and temporal responses of *B. napus* to *L. biglobosa*, particularly at integrated multi-omics levels, remain poorly understood. Here, we integrated physiological, transcriptomic, and proteomic analyses to characterize defense responses at 72, 120, and 168 h post inoculation (hpi). Physiological assays showed elevated peroxidase (POD), phenylalanine ammonia-lyase (PAL), polyphenol oxidase (PPO) activities, and malondialdehyde (MDA) content, indicating oxidative stress–associated defense activation. Multi-omics analyses revealed a temporally coordinated immune reprogramming process. At 72 hpi, pathogen perception, reactive oxygen species (ROS) accumulation, and metabolic adjustment dominated early responses. At 120 hpi, MAPK signaling and antioxidant systems, including glutathione S-transferases (GSTs), were strongly activated. At 168 hpi, phenylpropanoid biosynthesis, lignin deposition, and cell wall remodeling were enhanced, indicating structural reinforcement. Integrated transcriptome–proteome analysis identified key candidate regulators, including GSTs, caffeic acid O-methyltransferase (COMT), short-chain dehydrogenase/reductase (SDR) proteins, and MLP-like protein 28, showing coordinated and stage-specific expression patterns. Collectively, these results reveal a temporally ordered, multi-layered defense network in *B. napus*, characterized by sequential metabolic reprogramming, immune signaling activation, and structural reinforcement, providing mechanistic insights into *L. biglobosa* resistance.

## 1 Introduction

Oilseed rape (*Brassica napus.*), also known as rapeseed or canola, is one of the most important oilseed crops worldwide and serves as a major source of edible vegetable oil and industrial raw materials (Liu, 1984). In China, oilseed rape is the largest cultivated oil crop and plays an important role in ensuring edible oil security (Howlett, 2004; Wang et al., 2022). However, the productivity of oilseed rape is frequently threatened by various diseases, among which blackleg disease is considered one of the most destructive fungal diseases affecting rapeseed production worldwide (Li et al., 2008).

Blackleg disease is caused by fungal pathogens belonging to the genus Leptosphaeria and can result in premature senescence, stem cankers, lodging, and substantial yield losses. Based on pathogenicity, the causal agents are mainly classified into *Leptosphaeria maculans* and *Leptosphaeria biglobosa* (West et al., 2000). Among these species, *L. maculans* is generally regarded as the more aggressive pathogen and has therefore been the primary focus of most genetic, molecular, and omics-based studies on blackleg resistance. In contrast, *L. biglobosa* has traditionally been considered less virulent; however, increasing evidence suggests that it is widely distributed in many rapeseed-growing regions and can also cause significant yield losses under favorable environmental conditions (West et al., 2002; Khangura, 2004; Song et al., 2018; Zheng et al., 2020). Furthermore, accumulating studies indicate that *L. biglobosa* may trigger defense responses that differ from those induced by *L. maculans*. Rather than representing a simple variation in virulence, *L. biglobosa* infection may induce distinct and less-characterized immune regulatory programs, suggesting potential differences in host immune coordination compared with *L. maculans*.

Importantly, *L. biglobosa* is currently the predominant blackleg pathogen reported in China and has been detected across major rapeseed-producing regions (West et al., 2002; Hao, 2013; Song et al., 2018; Zheng et al., 2020). Despite its agricultural importance, the molecular mechanisms underlying *Brassica napus* responses to *L. biglobosa* infection remain poorly understood.

Plants have evolved sophisticated defense systems to resist pathogen invasion. The cell wall serves not only as the first physical barrier against pathogen penetration but also as an important platform for pathogen perception and immune signal transduction (Zhao and Dixon, 2014; Höfte and Voxeur, 2017; Vaahtera et al., 2019; Wan et al., 2021). Following pathogen recognition, plants activate multiple defense pathways, including pathogen-associated molecular pattern-triggered immunity (PTI), effector-triggered immunity (ETI), reactive oxygen species (ROS) bursts, phytohormone signaling, and secondary metabolite biosynthesis (Caplan et al., 2008; Zhang et al., 2016; Yang et al., 2019). These responses collectively contribute to pathogen restriction and host resistance.These pathways do not function independently but are dynamically interconnected in a stage-dependent manner, forming a coordinated regulatory network that integrates signaling, metabolism, and structural defense responses.

At the physiological level, several defense-related enzymes have been implicated in the response of *B. napus* to pathogen infection. Previous studies demonstrated that the activities of superoxide dismutase (SOD), phenylalanine ammonia-lyase (PAL), peroxidase (POD), and polyphenol oxidase (PPO) are associated with enhanced disease resistance (Wang et al., 2001; Jones and Dangl, 2006; Zuo et al., 2009; Miao, 2014; Song et al., 2015; Han et al., 2016; Liu et al., 2017). In addition, reactive oxygen species accumulation, membrane lipid peroxidation, and lignin deposition are important components of plant defense responses and contribute to the establishment of effective resistance against fungal pathogens (Wang et al., 2001; Zuo et al., 2009; Han et al., 2016; Liu et al., 2017).

Recent advances in high-throughput sequencing and proteomic technologies have greatly improved our understanding of blackleg resistance in Brassica species. Transcriptomic studies have revealed extensive transcriptional reprogramming involving MAPK signaling, reactive oxygen species (ROS) metabolism, phytohormone signaling, phenylpropanoid biosynthesis, and defense-related transcription factors during Leptosphaeria infection (Liu et al., 2018; Wang et al., 2021; Zhai et al., 2021). Dual RNA-seq analyses further demonstrated dynamic host–pathogen interactions during compatible and incompatible *Brassica–Leptosphaeria* interactions (Zhai et al., 2021). More recently, alternative splicing has been identified as an additional regulatory layer contributing to the defense response of *Brassica napus* against *L. biglobosa* infection, highlighting the complexity of transcriptional regulation (Padmathilake and Fernando, 2022; Li et al., 2021). At the proteomic level, resistance-associated proteins involved in antioxidant defense, pathogenesis-related responses, and secondary metabolism have been identified in blackleg-resistant rapeseed cultivars (Huang et al., 2019)Furthermore, integrative multi-omics studies combining transcriptomic, proteomic, metabolomic, and physiological datasets have demonstrated that plant defense responses are coordinated through interconnected regulatory networks rather than single-layer regulation (Liu et al., 2020; Cantila et al., 2023; Starosta et al., 2024; Ma et al., 2024).

Although these studies have substantially advanced our understanding of blackleg resistance, most investigations have focused on *L. maculans* and have primarily employed individual omics approaches. Consequently, integrated transcriptome–proteome analyses specifically addressing the temporal defense responses of *B. napus* to *L. biglobosa* remain scarce. Moreover, little is known about how transcriptomic and proteomic regulation are coordinated across different stages of infection, limiting our understanding of the molecular mechanisms underlying stage-specific resistance. Single-omics approaches are therefore insufficient to capture discrepancies between transcript accumulation and protein abundance, highlighting the necessity of integrated multi-omics strategies to resolve multilayered immune regulation.

However, how these defense pathways are temporally coordinated and hierarchically regulated across transcriptional and translational levels during *L. biglobosa* infection remains largely unknown, particularly in terms of stage-specific immune reprogramming and regulatory hierarchy.

Therefore, the major knowledge gaps include: (i) the lack of integrated transcriptome–proteome analyses during *L. biglobosa* infection; (ii) limited understanding of the temporal dynamics governing host defense responses; and (iii) insufficient identification of candidate regulators exhibiting coordinated transcript–protein expression patterns. Addressing these gaps is essential for elucidating the molecular basis of resistance to this economically important pathogen.

Therefore, in this study, we performed integrated physiological, transcriptomic, and proteomic analyses to characterize the dynamic defense responses of *B. napus* following *L. biglobosa* infection. By combining time-series transcriptome–proteome profiling with physiological assays at 72, 120, and 168 h post inoculation, we identified a stage-specific defense program characterized by early stress perception and metabolic reprogramming, intermediate activation of MAPK signaling and antioxidant defenses, and late reinforcement of phenylpropanoid metabolism and cell wall-associated responses. Furthermore, integrated analyses highlighted several candidate resistance-associated regulators, including glutathione *S-transferases* (GSTs), caffeic acid *O-methyltransferase* (COMT), short-chain dehydrogenase/reductase family proteins (SDRA), and *MLP-like protein 28*.

By integrating physiological measurements with time-resolved transcriptomic and proteomic datasets, this study advances beyond previous blackleg omics investigations by revealing the temporal coordination of defense responses and identifying candidate regulators exhibiting transcript–protein concordance during *L. biglobosa* infection.This work establishes a temporally resolved multi-omics framework that uncovers stage-specific immune reprogramming and regulatory coordination in *B. napus*, providing mechanistic insights into hierarchical defense regulation beyond single-layer omics studies.

These findings provide new insights into the temporal coordination of transcriptomic and proteomic responses during *L. biglobosa* infection and establish a comprehensive multi-omics framework for understanding blackleg resistance in *B. napus.*

## 2 Materials and methods

### 2.1 Plant growth and fungal treatment

**Figure 1:**
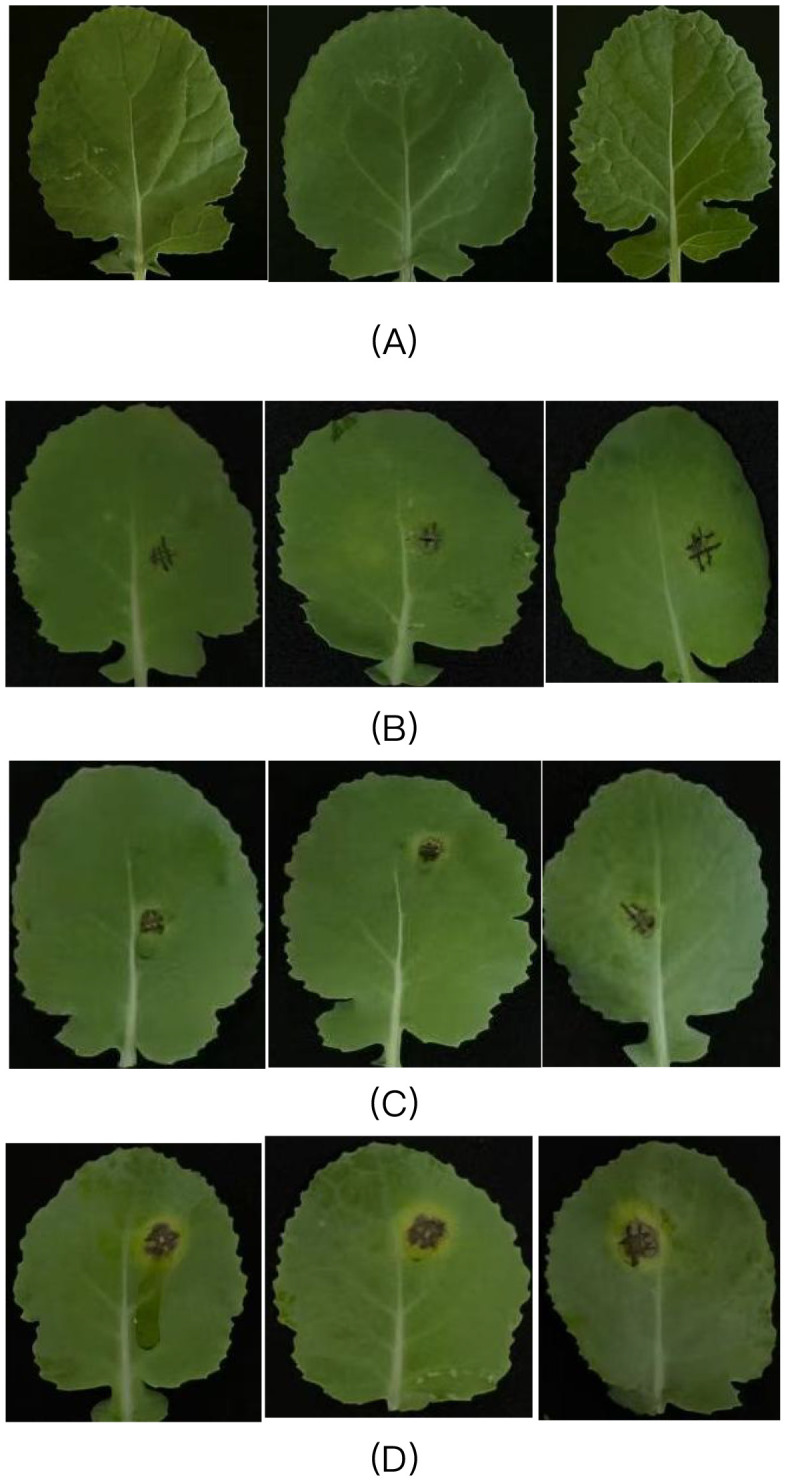
Phenotypic observation of rapeseed inoculated with *Leptosphaeria biglobosa*. **(A)** Rapeseed leaf tissue infected with *L. biglobosa*.for 0h; **(B)** Rapeseed leaf tissue infected with *L.biglobosa*. for 72h; **(C)** Rapeseed leaf tissue infected with *L. biglobosa*. for 120h; **(D)** Rapeseed leaf tissue infected with *L. biglobosa*. for 168h.

*Leptosphaeria biglobosa* NM-1, isolated and preserved in our laboratory, was used in this study. The strain was cultured on potato dextrose agar (PDA) at 25 °C for 10–15 days to induce spore production. Spores were eluted with sterile water, filtered through multiple layers of sterile gauze, and counted using a hemocytometer. The concentration of the spore suspension was adjusted to 1 × 10^7^ spores/mL for inoculation.NM88, a rapeseed variety selected in our laboratory for blackleg resistance, was used as the experimental material. Plants were grown in a climate-controlled greenhouse under a 25 °C/18 °C day/night regime, 40% relative humidity, and a 16 h light/8 h dark photoperiod.Six-leaf-stage seedlings were selected for inoculation. Prior to inoculation, a small puncture wound was made using a sterile needle.Plants designated as the baseline control were inoculated with 10 μL of sterile ddH_2_O and sampled immediately (0 h).The experimental plants were inoculated with 10 μL of a *L. biglobosa* spore suspension (1 × 10^7^ spores/mL) using a wound-assisted spot inoculation method.Inoculations were performed at the same position (the upper-right region of the first fully expanded leaf), with one inoculation site per plant. The inoculum concentration, inoculation volume, wound size, and inoculation position were kept consistent throughout the experiment.Samples from inoculated plants were collected at 72, 120, and 168 h post inoculation (hpi). The 0 h mock-treated samples were used as a single baseline reference to define infection-associated changes across all downstream analyses, and the study was designed to characterize infection-driven temporal responses rather than time-dependent developmental variation.Three biological replicates were included for each sampling point. Each biological replicate consisted of pooled tissues collected from three independent plants subjected to the same treatment, and equal amounts of tissue from the three plants were combined to generate one biological replicate.Disease symptom development was periodically documented photographically (**Figure 1**). Disease development was evaluated only through visual inspection and photographic documentation of symptom progression, successful infection was confirmed based on consistent symptom development at inoculation sites across biological replicates and sampling time points.

No quantitative disease severity scoring system or disease index calculation was applied in this study.Therefore, no disease severity index or quantitative scoring system was established in this study.

Samples from inoculated plants were collected at 72, 120, and 168 h post inoculation (hpi).

Sampling time points were selected based on preliminary observations of symptom progression and infection development.

At each sampling point, tissues were immediately frozen in liquid nitrogen and divided into two portions. One portion was stored at −80 °C for transcriptomic and proteomic analyses, whereas the other was used for enzyme activity assays and RT-qPCR validation.The same pooled biological samples were used for both transcriptomic and proteomic analyses to ensure direct comparability between transcriptome and proteome datasets.

### 2.2 Determination of physiological and biochemical indicators

To evaluate physiological changes in *B. napus* following inoculation with *L. biglobosa*, the activities of four key enzymes, peroxidase (POD), phenylalanine ammonia-lyase (PAL), polyphenol oxidase (PPO), and malondialdehyde (MDA), were measured at different time points. Enzyme activity assays were performed according to the manufacturer’s instructions using a commercial enzyme assay kit (Suzhou Greese Biotechnology Co., Ltd., Suzhou, China).Physiological assays were conducted using three independent biological replicates, and data are presented as the mean ± standard deviation (SD). The 0 h mock-treated samples served as the common baseline reference.

### 2.3 RNA-seq transcriptome sequencing

Total RNA was extracted from 12 samples using the RNAEastep^®^ SuperTotal RNA Extraction Kit (Promega Corporation, Madison, WI, USA) for RNA sequencing library construction. RNA degradation and contamination were assessed by 1% agarose gel electrophoresis, and RNA quality and concentration were evaluated using an Agilent 2100 Bioanalyzer (Agilent Technologies, Palo Alto, CA, USA) and a NanoDrop spectrophotometer (Thermo Fisher Scientific, Waltham, MA, USA). Sequencing was performed on an Illumina NovaSeq 6000 platform (Novogene, Wuhan, China). Raw sequencing data were filtered to obtain clean reads.

Clean reads were aligned to the Brassica napus ‘ZS11’ reference genome using HISAT2 (version 2.0.5) with default parameters. Transcript assembly and abundance estimation were performed using StringTie (version 1.3.3b), and read counts were generated using featureCounts (version 1.5.0-p3).

The RNA-seq dataset has been deposited in the NCBI Sequence Read Archive (SRA) under accession number PRJNA1475802.

Differentially expressed genes (DEGs) were identified using the DESeq2 package (version 1.20.0) in R. Genes with |log_2_(FoldChange)| ≥ 1 and adjusted P values (padj) ≤ 0.05 were considered significantly differentially expressed.

**Supplementary Table 2** has been expanded to include gene identifiers, functional annotations, log_2_ fold-change values across all time points, and adjusted P values (padj), enabling direct comparison of temporal transcriptional dynamics.

GO functional enrichment and KEGG pathway enrichment analyses were performed using the clusterProfiler package in R, with adjusted P values ≤ 0.05 considered statistically significant. Gene functional annotations, including GO terms and KEGG pathway mappings, were retrieved from the Brassica napus pan-genome information resource (BnPIR; http://cbi.hzau.edu.cn/bnapus/index.php).

### 2.4 Quantitative real-time PCR validation

To validate the reliability of the transcriptomic data, quantitative real-time PCR (qRT-PCR) was performed to analyze the expression levels of ten randomly selected differentially expressed genes. All primers were designed using Primer Premier 5.0 software and synthesized by The Beijing Genomics Institute (BGI, Beijing, China). First-strand cDNA was synthesized from total RNA using the PrimeScript™ RT Master Mix (Perfect Real Time; RR036A; TaKaRa, Dalian, China) according to the manufacturer’s instructions. qRT-PCR was performed using the QuantStudio™ 7 Flex real-time quantitative PCR system (Thermo Fisher Scientific, USA) and TB Green^®^ Premix Ex Taq™ II (Tli RNaseH Plus, Takara Code RR820A). β-actin was used as the internal reference gene. Each sample was analyzed in triplicate, including both biological and technical replicates. Relative gene expression levels were calculated using the 2^−ΔΔCt^ method.

### 2.5 Proteomic analysis

The same samples used for transcriptomic analysis were used for proteomic sequencing. Protein extraction, quantification, detection, and labeling were performed by Novogene (Wuhan, China) following standard protocols.

Database searching and protein quantification were conducted using Proteome Discoverer version 2.4 software.Proteins were identified using the BnPIR database (961441-zs11.all.v0.pep.fasta), and functional annotation was performed using the Gene Ontology (GO), Kyoto Encyclopedia of Genes and Genomes (KEGG), and Clusters of Orthologous Groups (COG) databases.

Differentially accumulated proteins (DAPs) were defined based on a fold change (FC) ≥ 1.20 or ≤ 0.83 and a P value < 0.05 (Student’s t-test). **Supplementary Table 1** has been expanded to include protein identifiers, functional annotations, fold change (FC) values at 72, 120, and 168 h post inoculation, statistical significance (P values), and temporal abundance patterns across infection stages.

GO annotation was performed using the Gene Ontology (GO) database, and KEGG enrichment analyses were subsequently conducted to characterize the biological functions of DAPs.

The raw and processed data have been deposited in iProX (PXD079373).

In addition, to ensure cross-omics comparability, annotation formats were standardized across transcriptomic and proteomic datasets. Specifically, proteomic data were reported as fold change (FC), whereas transcriptomic data were reported as log_2_ fold change (log_2_FC) due to differences in normalization strategies between the two platforms.

### 2.6 WGCNA analysis

Weighted gene co-expression network analysis (WGCNA) was performed using the WGCNA package in R. A soft-thresholding power of 12 was selected according to the scale-free topology criterion. Modules were identified using the dynamic tree cut algorithm with a minimum module size of 30 genes, and highly similar modules were merged using a cut height of 0.25.

### 2.7 STEM analysis

Temporal expression patterns of differentially expressed genes were analyzed using Short Time-series Expression Miner (STEM). The maximum number of model profiles was set to 50, and the maximum unit change between time points was set to 2. Profiles with P < 0.05 were considered significantly enriched.Statistical Analysis

### 2.8 Statistical analysis

Physiological assays, including peroxidase (POD), phenylalanine ammonia-lyase (PAL), polyphenol oxidase (PPO), and malondialdehyde (MDA) measurements, were conducted using three independent biological replicates. Data are presented as the mean ± standard deviation (SD). The 0 h mock-treated samples served as the common baseline reference, and differences among the 0, 72, 120, and 168 h sampling points were analyzed using one-way analysis of variance (ANOVA) followed by Tukey’s multiple comparison test. Statistical significance was defined as P < 0.05. All statistical analyses were performed using GraphPad Prism version 9.0 (GraphPad Software, San Diego, CA, USA).

## 3 Results

### 3.1 Physiological analyses

**Figure 2:**
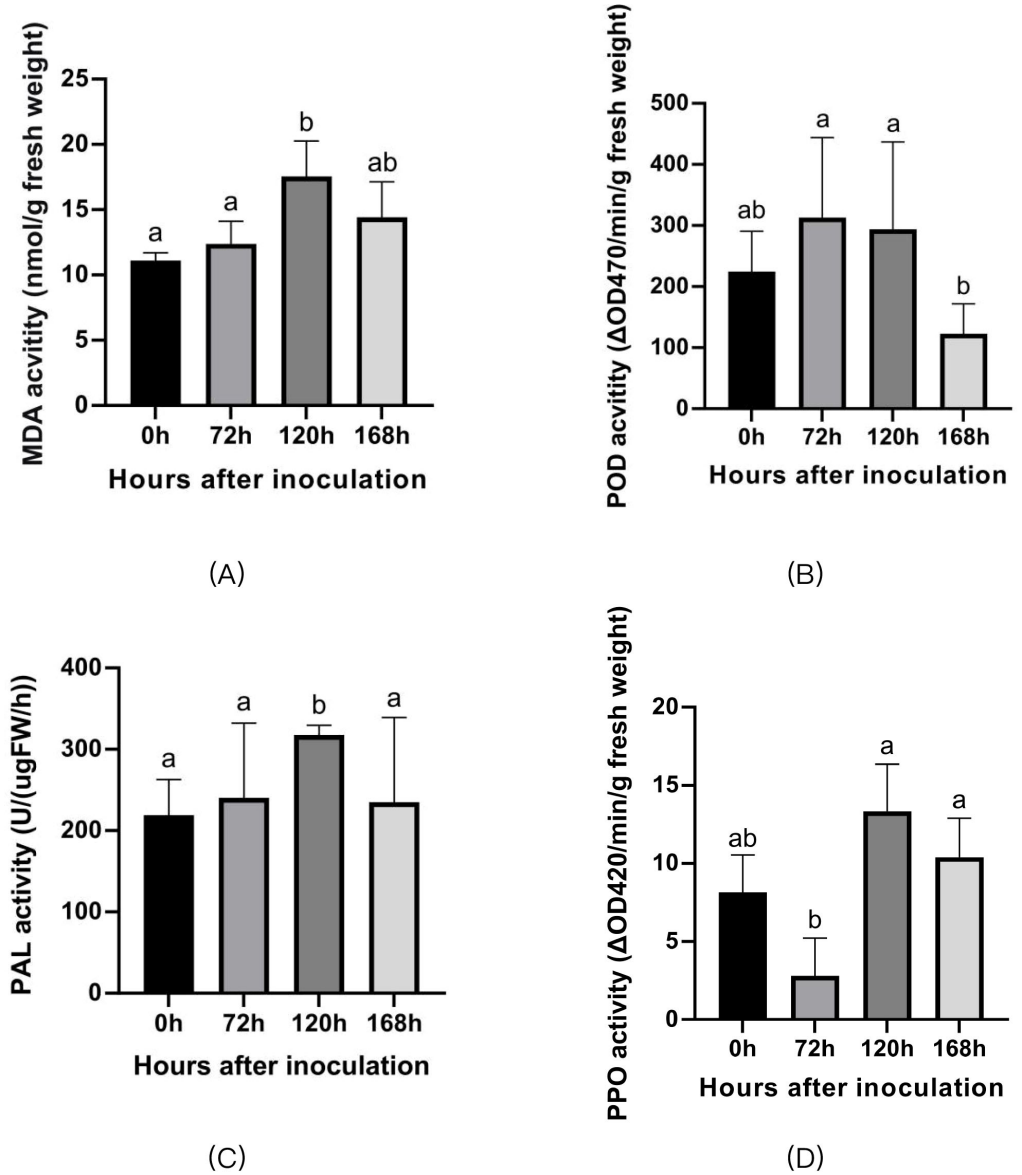
Changes in physiological indices of *Brassica napus* following inoculation with *Leptosphaeria biglobosa*.The horizontal axis represents the sampling time after inoculation (0, 72, 120, and 168 h post inoculation), and the vertical axis represents the corresponding physiological measurements. **(A)** Malondialdehyde (MDA) content. **(B)** Peroxidase (POD) activity. **(C)** Phenylalanine ammonia-lyase (PAL) activity. **(D)** Polyphenol oxidase (PPO) activity. Data are presented as the mean ± standard deviation (SD) of three biological replicates (n = 3). Different lowercase letters indicate statistically significant differences among sampling time points as determined by one-way analysis of variance (ANOVA) followed by Tukey’s multiple comparison test (P < 0.05).

To investigate the *in vivo* physiological responses of *Brassica napus*following infection with*L. biglobosa*, the activities of disease resistance–related enzymes (POD, PAL, and PPO) and the level of the membrane lipid peroxidation marker (MDA) were measured at different time points. Overall, these parameters exhibited dynamic changes during infection (**Figure 2**).

At 72 hpi, the activities of POD and PAL, as well as MDA content, increased compared with those at 0 hpi, whereas PPO activity decreased. At 120 hpi, POD, PAL, PPO, and MDA levels reached their peak. By 168 hpi, all indicators decreased significantly compared with those at 120 hpi and were not significantly different from those at 0 hpi.

### 3.2 Transcriptome sequencing and correlation analysis

In this study, transcriptome sequencing was performed on 12 samples of *Brassica napus* leaves collected at four time points (0, 72, 120, and 168 hpi) after blackleg infection. A total of 5.39 × 10^8^ raw reads were obtained (**Table 1**). After quality control, including the removal of low-quality reads, adapter contamination, and reads containing excessive unknown bases (N), a total of 5.20 × 10^8^ high-quality sequences (clean reads) were obtained. Among these, the proportion of reads with a quality score of Q20 exceeded 97% for all samples, whereas those with Q30 exceeded 93%. The GC content ranged from 46% to 47%, and the mapping rate of clean reads to the reference genome exceeded 85% for all samples. The Pearson correlation coefficients among the three biological replicates were greater than 0.9. The RNA-seq data have been deposited in the NCBI Sequence Read Archive (SRA) under accession number PRJNA1475802.

**Table 1:** Sequencing data statistics.

Sample	Raw_reads	Clean_reads	Clean_bases	Error_rate	Q20	Q30	GC_pct
0h_1	45854952	44605324	6.69G	0.03	97.42	92.84	47.78
0h_2	45559286	44349498	6.65G	0.03	97.59	93.24	47.59
0h_3	45317710	43549386	6.53G	0.03	97.71	93.54	47.67
72h_1	40761224	39495574	5.92G	0.03	97.58	93.19	47.16
72h_2	46017860	43666986	6.55G	0.03	97.48	93.03	47.32
72h_3	45411644	43634086	6.55G	0.03	97.45	92.92	46.82
120h_1	47226820	45580050	6.84G	0.03	97.68	93.44	46.7
120h_2	45873840	44334840	6.65G	0.03	97.61	93.25	46.93
120h_3	47282558	45775134	6.87G	0.03	97.57	93.19	47.06
168h_1	42227888	40793670	6.12G	0.03	97.58	93.21	46.73
168h_2	42297542	40847812	6.13G	0.03	97.67	93.38	46.71
168h_3	44745374	43637134	6.55G	0.03	97.43	92.85	46.51

1 Sample, sample analysis number; Raw reads, number of original reads; Clean reads, number of paired-end reads in the clean data; Clean bases, total number of bases; Error rate, overall sequencing error rate; Q20, percentage of bases with a quality score of 20 or higher; Q30, percentage of bases with a quality score of 30 or higher; GC content, percentage of G and C bases in the clean data.

All downstream transcriptomic analyses, including differential expression analysis, STEM clustering, and weighted gene co-expression network analysis (WGCNA), were performed using normalized gene expression matrices generated by DESeq2. Differentially expressed genes (|log_2_FoldChange| ≥ 1, adjusted P value ≤ 0.05) were used for downstream functional interpretation and candidate gene selection.

### 3.3 Analysis of differentially expressed genes

To analyze the transcriptional response of canola leaves following infection with Leptosphaeria biglobosa, differential expression gene (DEG) analysis was performed on samples collected at different time points. A total of 52, 965 DEGs were identified.

**Figure 3:**
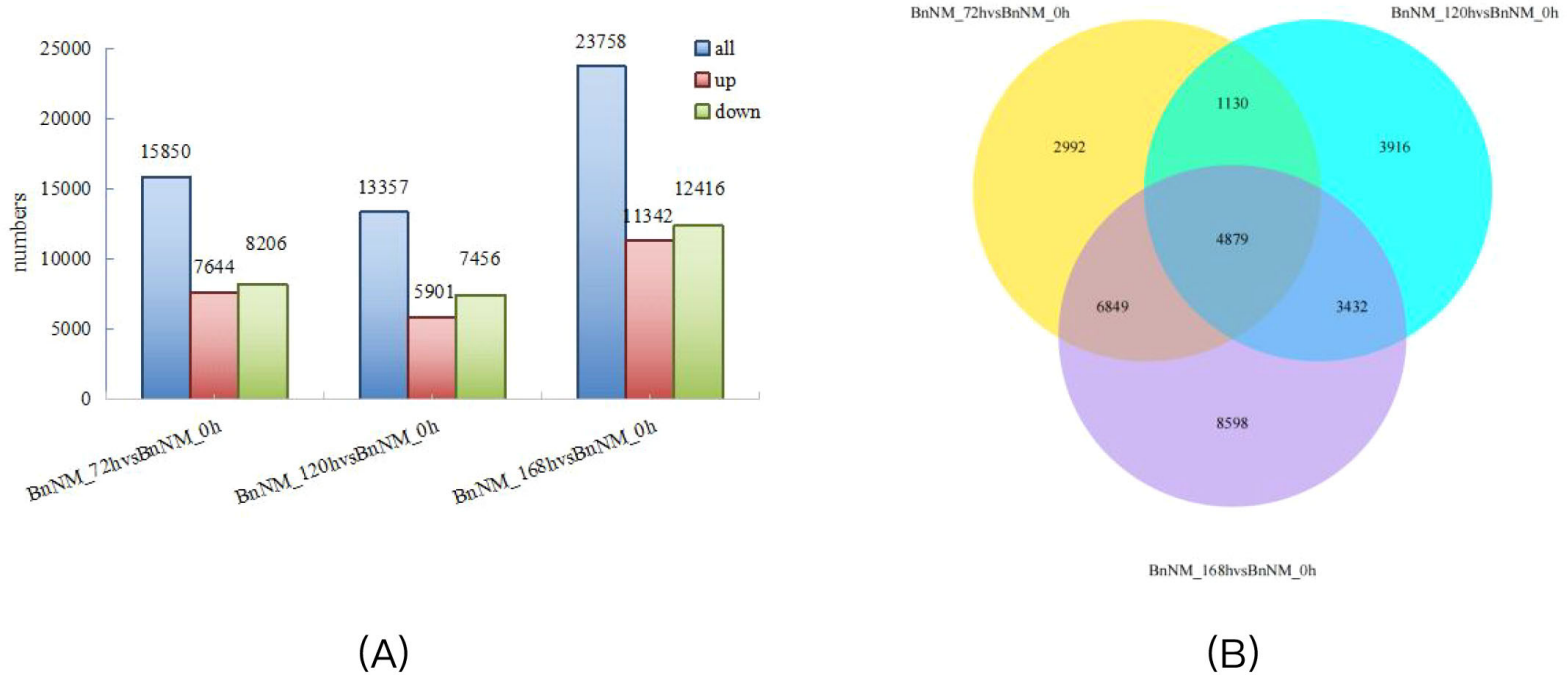
Differentially expressed genes (DEGs) in canola leaves infected by *Leptosphaeria biglobosa*. **(A)** Blue represents the total number of DEGs, red represents upregulation and green represents downregulation. **(B)** Overlap of differentially expressed genes across pairwise comparisons further highlighted shared and stage-specific transcriptional responses.

At 72 hpi compared with 0 hpi, 15, 850 DEGs were detected, including 7, 644 upregulated and 8, 206 downregulated genes. In the comparison between 120 and 0 hpi, 13, 357 DEGs were identified, of which 5, 901 were upregulated and 7, 456 were downregulated. At 168 hpi compared with 0 hpi, 23, 758 DEGs were detected, including 11, 342 upregulated and 12, 416 downregulated genes (**Figure 3A**).

Venn analysis of differentially expressed genes (DEGs) identified across pairwise transcriptomic comparisons revealed the overall distribution and shared transcriptional responses among infection stages (**Figure 3B**). A total of 15, 852 DEGs were identified across the comparisons, among which 4, 373 (44.33%) were specifically expressed during the 72–168 hpi period. Notably, 4, 879 DEGs exhibited sustained differential expression from 72 to 168 hpi, indicating a stable and prolonged transcriptional response during the mid-to-late infection stages. This pattern provides a global overview of DEG overlap dynamics and highlights the continuity of transcriptional reprogramming during L. biglobosa infection.

**Figure 4:**
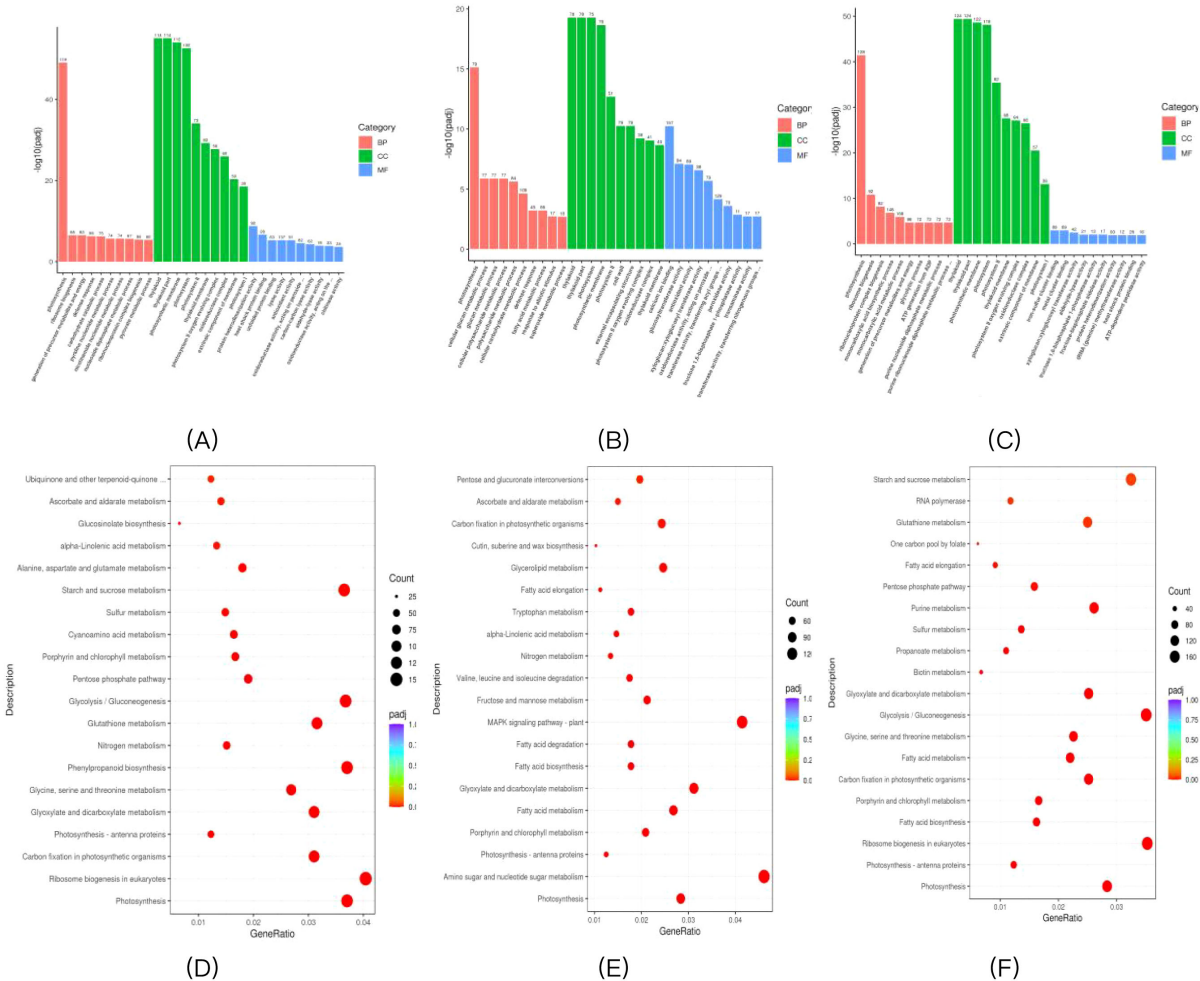
GO classification and KEGG enrichment analysis of deferential gene expressions (DEGs) in *Brassica napus* inoculated with *Leptosphaeria biglobosa*., sampled at 0, 72, 120and 160 hours post inoculation (hpi), **(A–C)**GO classification of DEGs, and **(D–F)** KEGG enrichment analysis of DEGs.

GO enrichment analysis of the DEGs (**Figures 4A–C**) showed that at 72 hpi, DEGs were mainly enriched in functional categories such as photosynthesis, ribosome biogenesis, energy metabolism-related processes (generation of precursor metabolites and energy), defense response, carbohydrate catabolism, and nicotinamide nucleotide metabolism. At 120 hpi, DEGs were mainly enriched in cell wall organization, lignin metabolic processes, defense response, extracellular regions, cell wall components, oxidoreductase activity, and chitinase activity. At 168 hpi, DEGs were primarily enriched in biological processes such as photosynthesis, ribosome biogenesis, ribonucleoprotein complex biogenesis, and monocarboxylic acid biosynthesis. At the cellular component level, enrichment was mainly observed in thylakoids, thylakoid components, photosynthetic membranes, and photosystems. At the molecular function level, enrichment was mainly associated with iron–sulfur cluster binding, metal cluster binding, and xyloglucan:xyloglucosyl transferase activity.

KEGG enrichment analysis (**Figures 4D–F**) showed that the DEGs were involved in multiple pathways, including glucosinolate biosynthesis and the MAPK signaling pathway. In the glucosinolate biosynthesis pathway, *CYP79B2* (BnaA01G0008700ZS) and *SOT17* (BnaA06G0128100ZS) were upregulated following pathogen infection.

### 3.4 STEM and KEGG analysis of DEGs

5, 852 DEGs were classified into six distinct temporal expression clusters by STEM time-series analysis.

Clusters 1, 2, and 3 contained 871, 1, 023, and 1, 165 DEGs, respectively. Overall, the expression levels of genes in these clusters showed a decreasing trend. Although some genes were transiently upregulated at 72 hpi, their expression gradually declined over time. KEGG enrichment analysis (*P* < 0.05) indicated that these genes were mainly involved in photosynthesis, fatty acid synthesis and metabolism, amino acid degradation, peroxisomal function, and secondary metabolite biosynthesis.

Cluster 4 contained 584 DEGs that were upregulated at 72 hpi, downregulated at 120 hpi, and re-upregulated at 168 hpi. These genes were mainly enriched in pathways related to thiohydroxamate biosynthesis, sulfur metabolism, and amino acid metabolism.

Cluster 5 consisted of 708 DEGs, which showed a continuous increase in expression and were mainly associated with carbohydrate metabolism, RNA degradation, and α-linolenic acid metabolism pathways.

**Figure 5:**
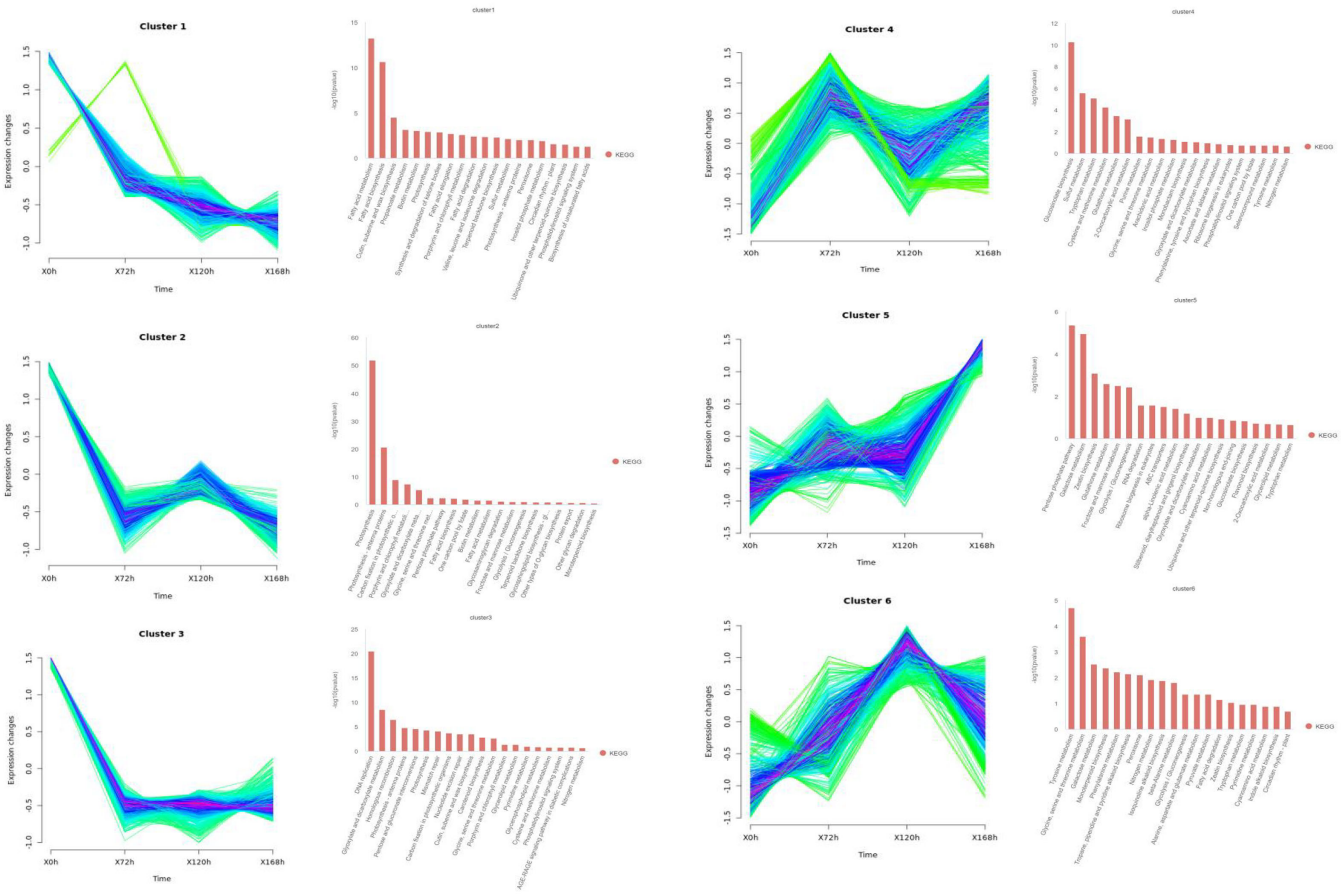
Time-series transcriptomics analysis of DEGs in response to *L. biglobosa* infection. Left: STEM analysis of DEGs. Right: KEGG pathways enrichment of DEGs in each cluster.

DEGs in cluster 6 were upregulated at 72 hpi, peaked at 120 hpi, and then gradually decreased. These genes were mainly enriched in pathways related to amino acid metabolism, alkaloid biosynthesis, and peroxisome signaling (**Figure 5**).

### 3.5 WGCNA analysis of DEGs

**Figure 6:**
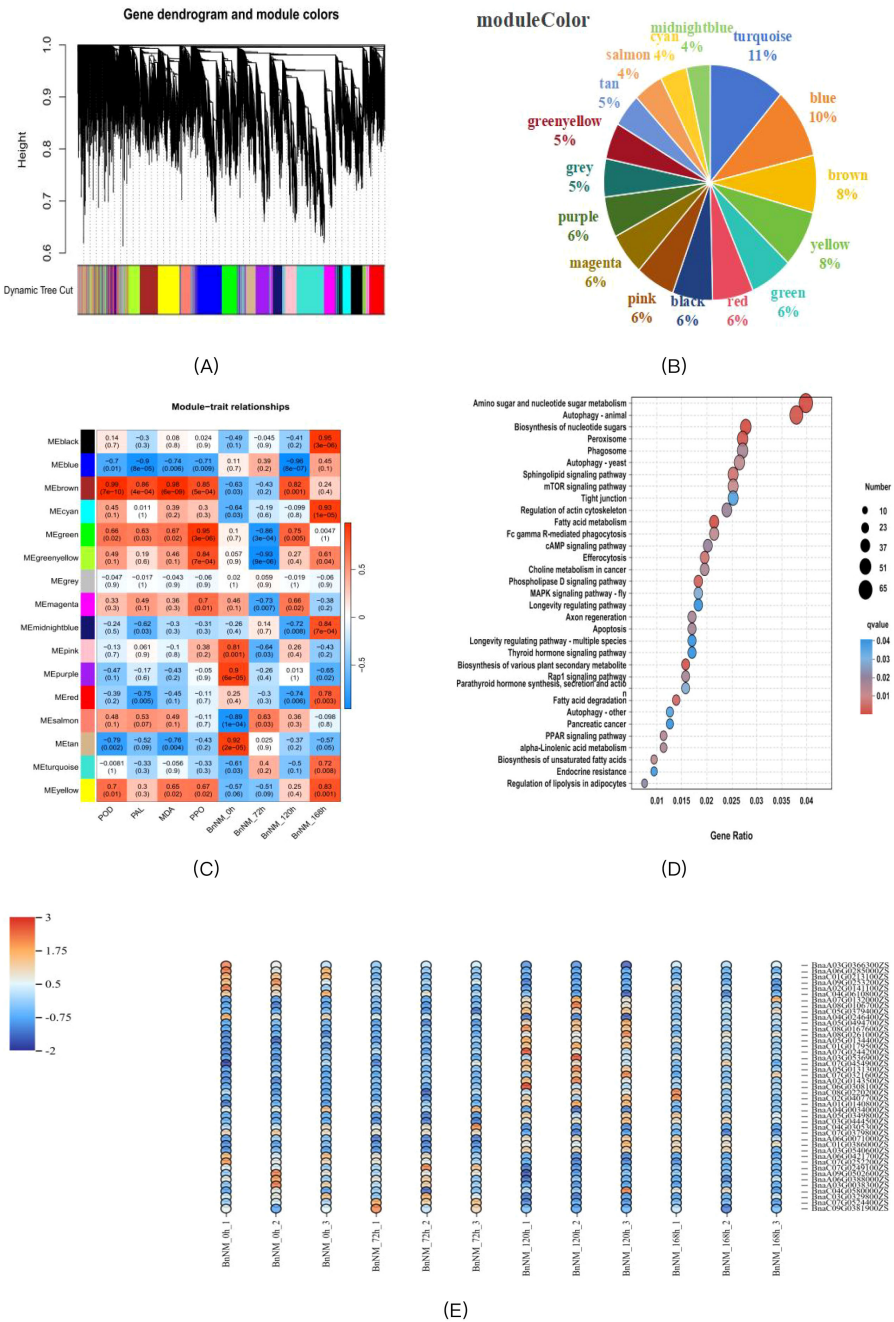
Weight gene co-expression network analysis (WGCNA) plot. **(A, B)** Module Statistics Pie chart. **(C)** Heat map of correlation between modules, traits and groups. **(D)** KEGG annotation of MEbrown module with DEGs profile. **(E)** Heatmap of DEGs enrinched in MAPK signaling pathway.

15, 852 DEGs were subjected to WGCNA, resulting in 16 co-expression modules (**Figure 6A**).

The turquoise module contained the largest number of genes (4, 718 genes, 11% of total), whereas the midnightblue module contained the fewest (1, 467 genes) (**Figure 6B**).

Correlation analysis between module eigengenes, defense-related traits (POD, PAL, PPO, and MDA), and infection stages (72, 120, and 168 hpi) identified three key modules (MEbrown, MEblack, and MEgreenyellow) with strong associations (correlation coefficient > 0.8) (**Figure 6C**).

Among these, the MEbrown module showed the strongest positive correlation with POD, PAL, MDA, and samples at 120 hpi. Functional enrichment analysis revealed that genes in this module were mainly associated with MAPK signaling, secondary metabolite biosynthesis, peroxisome function, metabolic processes, and hormone-related regulation (**Figure 6D**). Notably, 43 genes within this module were enriched in the MAPK signaling pathway (**Figure 6E**).

The identification of MAPK signaling as a central hub in both transcriptomic and proteomic datasets further supports its role as a key regulatory module coordinating early immune responses.

### 3.6 Proteome sequencing

TMT-labeled quantitative proteomics was used to analyze changes in protein expression in rapeseed following infection with blackleg disease.

Protein identification and quantification were performed using Proteome Discoverer software (version 2.4) following standard parameters provided by Novogene. The coefficient of variation (CV) analysis of all quantified proteins (**Supplementary Figure 2**) indicated good reproducibility and supported the reliability of the dataset.

The raw mass spectrometry data have been deposited in the iProX repository under accession number PXD079373.

A total of 57, 909 peptides corresponding to 10, 174 proteins were identified from 12 samples, of which 10, 122 proteins were quantified.

Differentially expressed proteins (DEPs) were defined based on fold change (FC ≥ 1.20 or ≤ 0.83) and a raw P value < 0.05.

**Figure 7:**
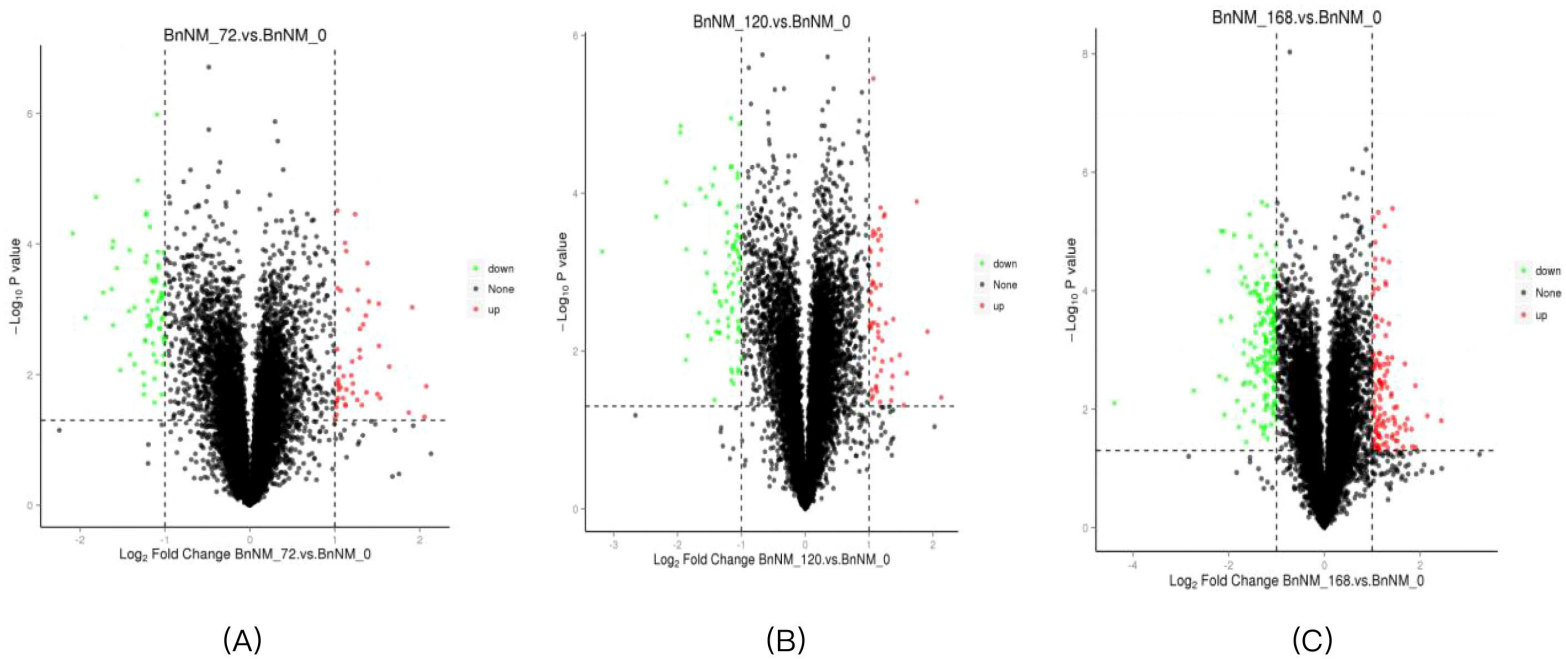
Volcano plots of differentially expressed proteins (DEPs) in BnNM cells at different time points after treatment. **(A)** 72 h vs. 0 h; **(B)** 120 h vs. 0 h; **(C)** 168 h vs. 0 h. Each point represents an individual protein. The x-axis indicates log_2_ fold change, and the y-axis indicates −log_10_(P-value). Red and green dots represent significantly upregulated and downregulated proteins, respectively (|log_2_FC| ≥ 1 and P < 0.05), while black dots indicate proteins with no significant change.

At 72 h post-inoculation compared with 0 h, 114 DEPs were identified in canola leaves, Venn analysis of differentially accumulated proteins (DAPs) across infection stages revealed a similar pattern of temporal divergence at the proteome level. Although a subset of proteins was shared among stages, a large proportion exhibited stage-specific accumulation patterns, reflecting dynamic proteomic reprogramming during host–pathogen interaction.

including 49 upregulated and 65 downregulated proteins. At 120 h post-inoculation, 138 DEPs were identified (56 upregulated and 82 downregulated), and at 168 h post-inoculation, 274 DEPs were detected (117 upregulated and 157 downregulated) (**Figure 7**).

### 3.7 Analysis of differentially expressed proteins

**Figure 8:**
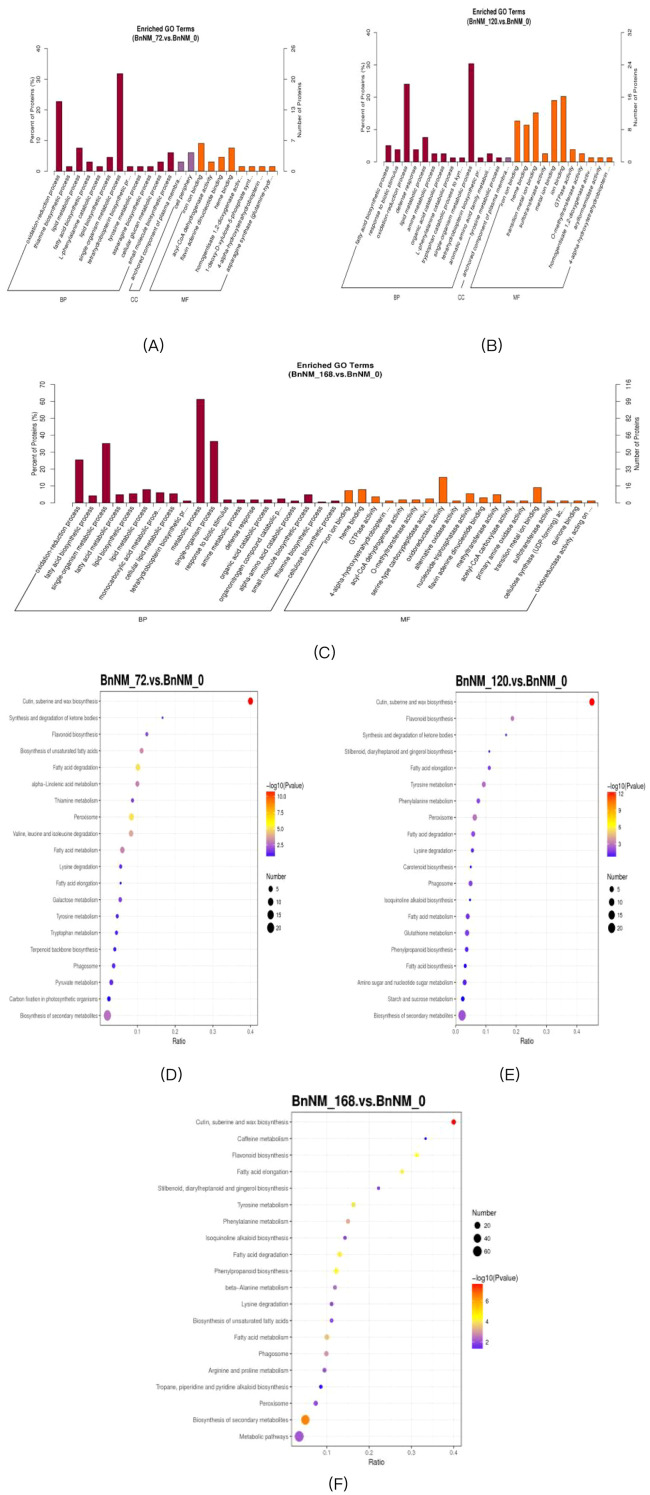
GO classification and KEGG enrichment analysis of deferential proteins expressions (DEGs) in Brassica napus inoculated with Leptosphaeria biglobosa., sampled at 0, 72, 120and 168 hours post inoculation (hpi), **(A–C)**GO classification of DEPs, and **(D–F)** KEGG enrichment analysis of DEPs.

GO enrichment analysis (**Figures 8A–C**) showed that at 72 hpi, DEPs were mainly associated with redox processes (GO:0055114), iron ion binding (GO:0005506), acyl-CoA dehydrogenase activity (GO:0003995), thiamine biosynthesis (GO:0009228), lipid metabolic process (GO:0006629), heme binding (GO:0020037), and plasma membrane-anchored components (GO:0046658).

At 120 hpi, DEPs were enriched in redox processes, fatty acid biosynthesis, response to biotic stimulus (GO:0009607), defense response (GO:0006952), and single-organism metabolic processes (GO:0044710). Enriched molecular functions included iron ion binding, heme binding, and transition metal ion binding, while cellular components were mainly associated with the cell periphery, cell wall (GO:0005618), and apoplast (GO:0048046).

At 168 hpi, DEPs were enriched in redox processes, fatty acid and lipid metabolism, and monocarboxylic acid metabolic processes. Molecular functions were mainly related to iron ion binding, heme binding, and oxidoreductase activity (GO:0016491), while cellular components were associated with membrane and integral membrane components.

KEGG enrichment analysis (**Figures 8D–F**) showed that at 72 hpi, DEPs were mainly involved in biosynthesis of secondary metabolites (map01110), cutin, suberine and wax biosynthesis (map00073), peroxisome (map04146), fatty acid degradation (map00071), flavonoid biosynthesis (map00941), and unsaturated fatty acid biosynthesis (map01040). At 120 and 168 hpi, DEPs were further enriched in cutin, suberine and wax biosynthesis, phenylpropanoid biosynthesis (map00940), fatty acid metabolism (map00071; map00062), and secondary metabolite biosynthesis.

### 3.8 Correlation analysis of transcriptome and proteome sequencing

Only a subset of DEGs exhibited corresponding changes at the protein level due to the limited coverage of TMT-based proteomics and post-transcriptional regulation effects.

To further resolve stage-dependent transcriptional reprogramming at the systems level, a stage-wise Venn analysis of DEGs across 72, 120, and 168 hpi was performed.This analysis highlights progressively differentiated gene sets underlying temporal specialization of host responses.

To obtain a system-level view of host responses, we integrated transcriptomic and proteomic datasets across all infection stages (0, 72, 120, and 168 hpi) by jointly analyzing DEGs and DAPs. This integrated analysis revealed both conserved and highly stage-specific molecular responses during disease progression.

**Figure 9:**
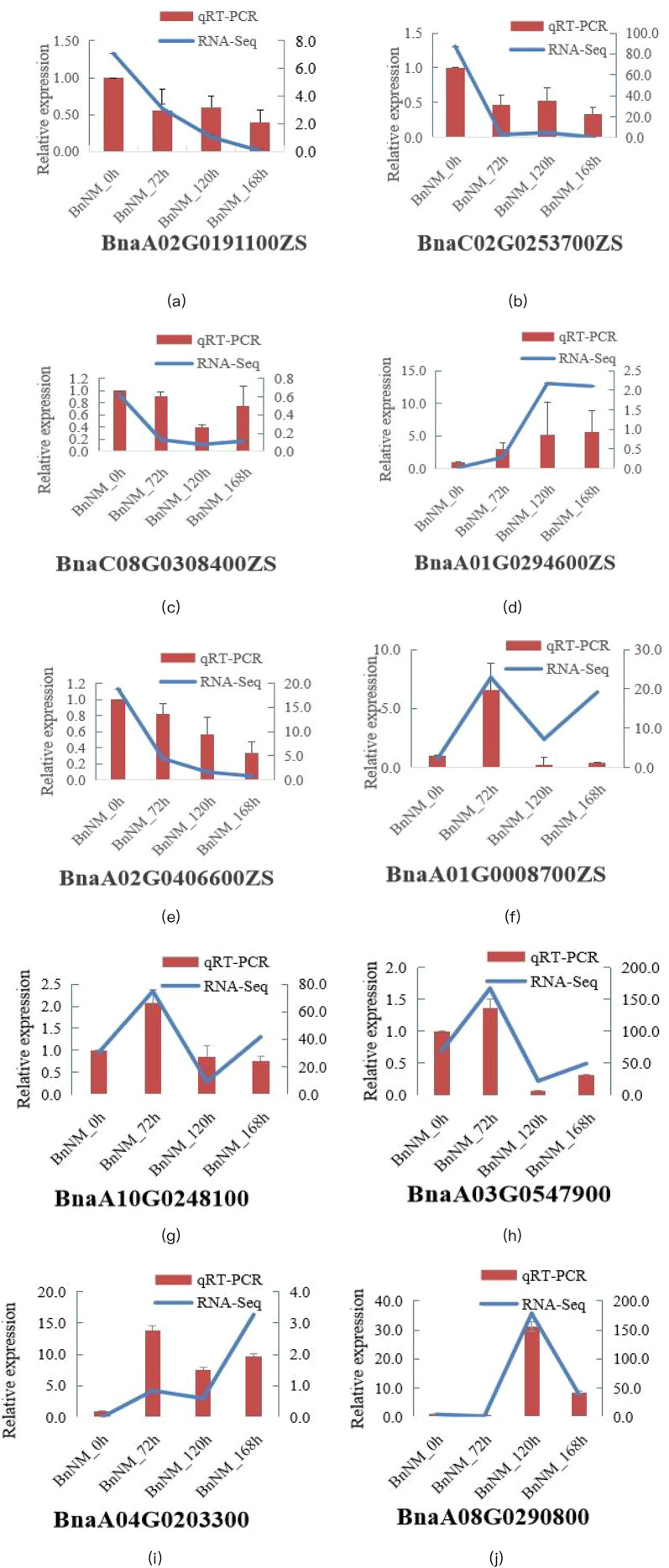
Integrated multi-omics analysis of temporal defense responses in *Brassica napus* during *Leptosphaeria biglobosainfection*. **(a)** Venn diagram showing overlap of differentially expressed genes (DEGs) across 72, 120, and 168 h post-inoculation (hpi). **(b)** Venn diagram showing overlap of differentially accumulated proteins (DAPs) across the same time points. **(c)** Integrated heatmap of representative defense-related genes and corresponding proteins. **(d)** Clustered heatmap of representative differentially accumulated proteins involved in major defense pathways. **(e)** Proposed stage-specific defense model of *B. napus* during infection. Expression values in heatmaps are shown as Z-scores (blue to red scale), indicating relative abundance changes across stages.

Venn diagram analysis of differentially expressed genes (DEGs) across infection stages revealed an increasing divergence of transcriptional responses over time, with a small subset of genes consistently regulated across all stages and a large proportion exhibiting stage-specific expression patterns (**Figure 9A****).**

Venn diagram analysis of differentially accumulated proteins (DAPs) showed a similar pattern at the proteome level, characterized by progressive temporal divergence and pronounced stage specificity, although a subset of proteins remained shared among multiple infection stages (**Figure 9B**).

Overall, Venn analysis showed an increasing divergence of both DEGs and DAPs over time, indicating progressive temporal reprogramming of the host response. While a small core set of genes and proteins remained consistently regulated across all stages, most components exhibited strong stage specificity, reflecting dynamic reorganization of defense strategies during infection.

Clustered heatmap analysis further resolved these temporal patterns, revealing coordinated gene–protein modules associated with early stress perception (72 hpi), immune signaling activation (120 hpi), and structural reinforcement (168 hpi). Although transcript and protein levels showed partial concordance, a substantial number of components exhibited stage-dependent discordance, suggesting post-transcriptional regulation as an additional layer of immune control.Integrated visualization of representative genes and their corresponding proteins revealed coordinated as well as discordant expression modules across infection stages (**Figure 9C**), further supporting post-transcriptional regulation as an additional layer shaping immune responses.These stage-specific molecular patterns suggest a progressive shift from metabolic adjustment to signaling activation and finally structural defense reinforcement.

Pairwise comparisons further supported these trends, with 40, 60, and 115 genes showing concordant transcript–protein changes at 72, 120, and 168 hpi, respectively.

GO enrichment of these shared components showed a progressive expansion of functional categories from primary metabolism at early stages to defense response, biosynthesis, and stress adaptation at later stages (**Supplementary Figure 3**). KEGG analysis consistently highlighted enrichment in cutin, suberine and wax biosynthesis, phenylpropanoid biosynthesis, fatty acid metabolism, and secondary metabolite biosynthesis across all time points, reinforcing the central role of metabolic reprogramming in host defense.

Together, these results demonstrate a tightly coordinated temporal coupling between transcriptomic and proteomic regulation, providing a unified systems-level framework of stage-specific immune reprogramming in Brassica napus.

#### 3.8.1 Proteins in the plant–pathogen interaction pathway

In the proteomic analysis, 11 differentially expressed proteins (DEPs) associated with plant–pathogen interaction pathways were identified, and these proteins were also detected at the transcriptomic level.

In the 120 hpi group, two defense-related proteins (MLP-like protein 28) were downregulated, whereas two glutathione S-transferase (GST) family proteins were upregulated. One peroxisome-related protein (long-chain acyl-CoA synthetase 2) was downregulated, whereas another protein, short-chain dehydrogenase/reductase (SDRA), was upregulated.

In addition, several proteins involved in cutin, suberin, and wax biosynthesis (e.g., *ECERIFERUM 1*, fatty acyl-CoA reductase 3, and alkane hydroxylase MAH1) were significantly downregulated at 120 h group. Some proteins involved in secondary metabolism (such as 3-ketoacyl-CoA synthase, phytoene synthase, and transaldolase 2) showed mixed expression patterns, with both upregulated and downregulated proteins observed.

#### 3.8.2 Proteins in the MAPK signaling pathway

Based on integrated transcriptomic and proteomic datasets, proteins mapped to the MAPK signaling pathway were further examined, 20 DEPs were identified in the MAPK signaling pathway associated with plant–pathogen interactions.

At 72 h, four DEPs were identified, including three upregulated and one downregulated protein. At 120 hpi, nine DEPs were detected. Among these, three belonged to the glutathione S-transferase (GST) family, two were enzymes involved in tyrosine metabolism (primary amine oxidase and homogentisate 1, 2-dioxygenase), and two were peroxisome-related proteins (short-chain dehydrogenase/reductase, SDRA), all of which were upregulated. In addition, two MLP-like protein 28 proteins associated with defense responses were downregulated.

At 168 hpi, seven DEPs were identified. Among these, two belonged to the glutathione S-transferase (GST) family, two were enzymes involved in flavonoid biosynthesis (caffeoyl-CoA O-methyltransferase), and one was a peroxisome-related protein (short-chain dehydrogenase/reductase, SDRA), all of which were upregulated. In addition, two MLP-like protein 28 proteins were downregulated. The remaining proteins were associated with downstream components of the MAPK signaling pathway.

### 3.9 DEG profiling validation by qRT-PCR analysis

**Figure 10:**
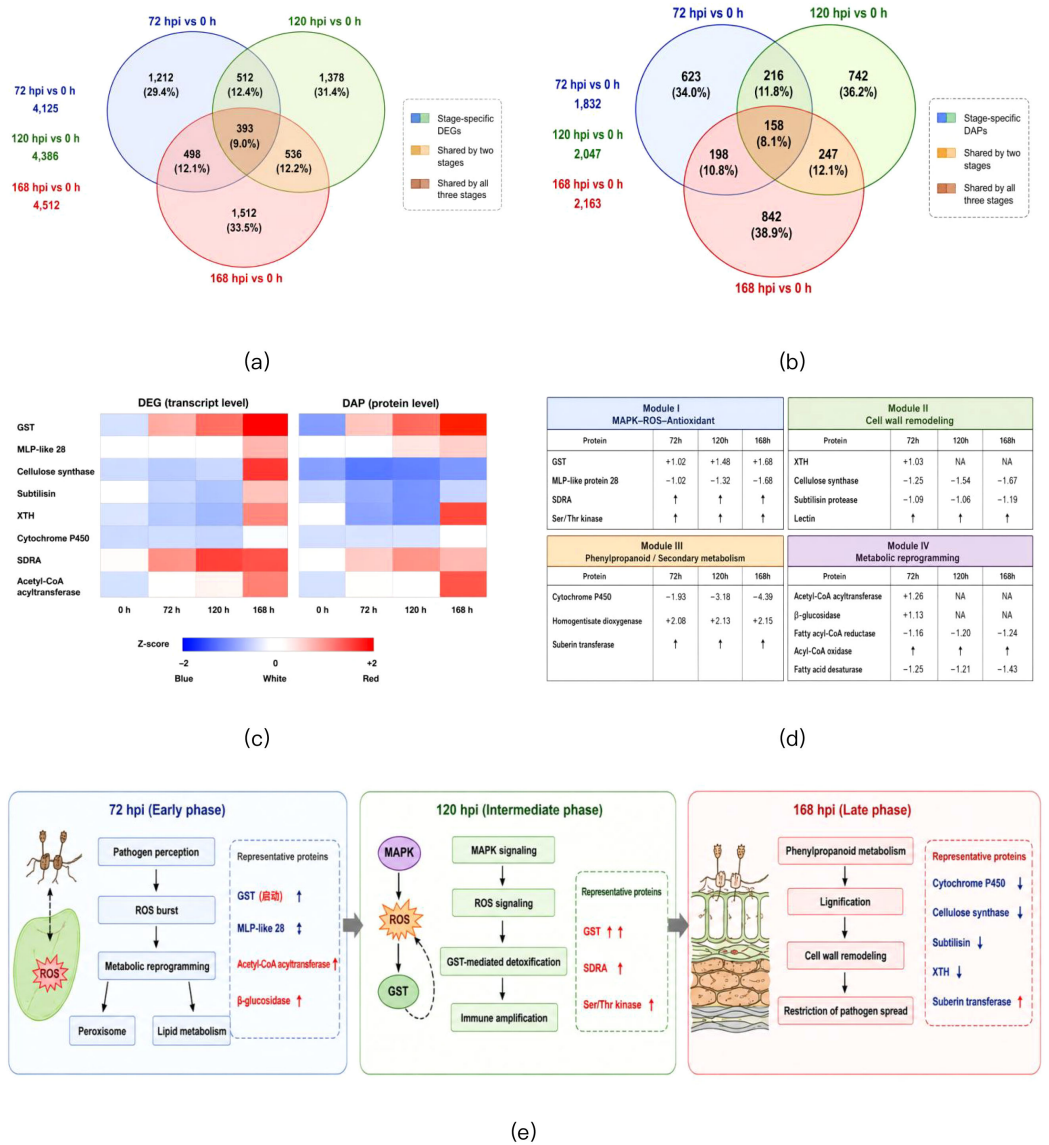
Validation of differentially expressed genes in canola leaves infected by *Leptosphaeria biglobosa* using quantitative polymerase chain reaction (qPCR). Sampling time points included 0 h, 72 h, 120 h, and 168 h. Red bars represent qRT-PCR results, and blue lines represent RNA-Seq results for the following genes: **(a)** BnaA03g12450D; **(b)** BnaA09g05640D; **(c)** BnaA10g06480D; **(d)** BnaC02g12570D; **(e)** BnaC04g02840D; **(f)** BnaC04g48390D; **(g)** BnaC06g34420D; **(h)** BnaC07g33180D; **(i)** BnaC07g45100D; **(j)** BnaC08g14940D. Relative expression levels of each gene were normalized to the 0 h time point (set to 1.0 or corresponding calibrated value), showing consistent trends and some discrepancies across time points. qRT-PCR data are presented as means (error bars indicate standard deviation), and RNA-Seq data are shown as FPKM or normalized read counts.

The expression levels of 10 genes associated with blackleg disease resistance in rapeseed were analyzed using quantitative real-time PCR (qRT-PCR). The results showed that the qRT-PCR expression patterns of these 10 differentially expressed genes (DEGs) were consistent with those obtained from the transcriptome data (FPKM values), confirming the reliability of the transcriptome analysis (**Figure 10**).

### 3.10 Temporal dynamics of representative defense-associated proteins

A focused analysis of representative defense-associated proteins involved in antioxidant regulation, metabolic reprogramming, phenylpropanoid metabolism, and cell wall remodeling revealed distinct stage-dependent expression patterns (**Figure 9D****).** GST and tetraspanin proteins exhibited sustained upregulation throughout infection, whereas MLP-like protein 28, cytochrome P450, cellulose synthase, and subtilisin-like protease showed progressive downregulation. Proteins involved in cell wall modification, including XTH, displayed transient induction during early infection. Collectively, these patterns support a sequential defense strategy characterized by early stress responses, intermediate antioxidant regulation, and late structural reinforcement.These multi-omics datasets collectively suggest a progressive and coordinated reprogramming of host defense across transcriptional and translational levels.

### 3.11 Integrated stage-specific defense model of Brassica napus during L. biglobosa infection

To integrate the multi-omics evidence into a coherent biological framework, the temporal molecular responses were summarized into a stage-specific defense model (**Figure 9E****).** At the early stage (72 hpi), pathogen perception was accompanied by ROS accumulation and rapid metabolic reprogramming, reflecting an initial stress response dominated by energy redistribution and redox imbalance. At the intermediate stage (120 hpi), activation of MAPK signaling and glutathione S-transferase (GST)-mediated detoxification formed a central regulatory hub coordinating immune signaling and ROS homeostasis. At the late stage (168 hpi), phenylpropanoid metabolism and cell wall remodeling were strongly activated, leading to lignification and reinforcement of structural barriers against pathogen invasion.

This model provides a unified systems-level framework for blackleg resistance in Brassica napus during L. biglobosa infection.

## 4 Discussion

Over time, plants have evolved sophisticated defense systems against pathogen invasion, in which the cell wall functions both as a physical barrier and as a platform for immune perception and signal transduction (Höfte and Voxeur, 2017; Vaahtera et al., 2019; Wan et al., 2021). Plant immune responses are initiated through danger perception systems that recognize pathogen-associated signals and activate downstream defense signaling cascades (Jones and Dangl, 2006; Dodds and Rathjen, 2010; Zhou and Zhang, 2020). In this study, genes and proteins were strongly enriched in processes related to cell wall organization, lignin metabolism, and chitinase activity during the mid-to-late infection stages (120–168 hpi). Persistent enrichment of phenylpropanoid biosynthesis and cutin, suberin, and wax biosynthesis indicates that cell wall remodeling is a core component of rapeseed resistance to *L. biglobosa*. Cuticle and cell wall reinforcement act as critical structural barriers against pathogen penetration (Serrano et al., 2014). Phenylpropanoid metabolism contributes to structural reinforcement through lignin deposition, and its coordinated transcriptional and proteomic regulation supports its central role in defense, consistent with previous functional evidence of C4H (Zhao and Dixon, 2014; Dixon and Barros, 2019). Flavonoid and phenylpropanoid derivatives also participate in defense-related secondary metabolism, further reinforcing stress tolerance (Ferreyra et al., 2012).

Early infection (72 hpi) was characterized by suppression of photosynthesis and energy metabolism, together with activation of peroxisomal pathways and lipid degradation (Jones and Dangl, 2006; Mittler, 2017; Zhou and Zhang, 2020). These changes reflect a rapid metabolic shift that supports ROS-mediated signaling during early defense priming. Such metabolic reprogramming is consistent with a general plant–pathogen interaction framework in which primary metabolism is reallocated toward immune activation (Chen et al., 2020).

At 120 hpi, MAPK signaling emerged as a central regulatory hub that integrates early ROS signals with downstream transcriptional reprogramming and metabolic defense activation (Dixon and Paiva, 1995; Zhang and Klessig, 2001; Asai et al., 2002; Meng and Zhang, 2013; Zipfel, 2014).Within this framework, ROS homeostasis was tightly regulated through glutathione S-transferase (GST)-mediated detoxification, ensuring a balance between defense signaling and oxidative damage control (Gullner et al., 2018). Together, these processes constitute a coordinated MAPK–ROS–GST regulatory module that links signal perception to redox buffering and immune amplification, thereby preventing excessive cellular injury while sustaining defense activation.

Carbohydrate and lipid metabolism were strongly reprogrammed during infection, supporting energy redistribution toward defense processes (Xiao et al., 2000; Proels and Hückelhoven, 2014). These pathways also contribute to defense signaling and membrane remodeling (Gullner et al., 2018).

Integrated multi-omics analysis identified key regulators including GST, SDRA, COMT, and MLP-like protein 28. GST and SDRA participate in redox regulation and lipid metabolism, respectively (Gullner et al., 2018; Azevedo et al., 2021). COMT is involved in lignin biosynthesis, while MLP-like protein 28 is suggested to act as a negative regulator of plant defense responses (Chen et al., 2020).These regulators may act as key coordination nodes linking transcriptional reprogramming with metabolic and structural defense responses during infection.

Based on these findings, a temporal defense model is proposed in which early ROS signaling initiates metabolic reprogramming, followed by MAPK-mediated immune activation and antioxidant regulation, and finally phenylpropanoid-driven cell wall reinforcement (Jones and Dangl, 2006; Meng and Zhang, 2013; Zipfel, 2014) (**Figure 9****).**

Notably, compared with previously reported responses to *L. maculans*, *L. biglobosa* infection appears to induce a more gradually phased and temporally coordinated activation of immune and metabolic pathways, suggesting pathogen-specific modulation of host signaling dynamics.

Transcript–protein concordance was moderate, likely due to post-transcriptional regulation and limited proteome coverage, consistent with previous multi-omics studies (Buccitelli and Selbach, 2020; Cantila et al., 2023). Additionally, the limited depth of proteomic coverage and the absence of pathogen-level quantification (e.g., fungal biomass assessment) may have constrained the resolution of host–pathogen interaction dynamics.

In addition, the absence of a quantitative disease severity scoring system represents a methodological limitation, as phenotypic evaluation in this study was based solely on visual symptom observation. Moreover, no time-matched mock controls were included at 72, 120, and 168 h, and the 0 h samples were used as the sole baseline reference. Future studies incorporating standardized disease scoring, pathogen biomass quantification (e.g., qPCR-based fungal load), and microscopic validation of fungal colonization will provide a more comprehensive and robust evaluation of infection dynamics and host resistance.

## 5 Conclusions

This study provides an integrated transcriptomic and proteomic analysis of *Brassica napus* in response to *Leptosphaeria biglobosa* infection, revealing a temporally coordinated defense architecture underlying host–pathogen interactions.

The results demonstrate that *B. napus* mounts a stage-specific immune strategy characterized by early ROS-associated metabolic reprogramming, followed by MAPK-mediated immune signal integration and antioxidant regulation, and culminating in phenylpropanoid-driven cell wall reinforcement. This coordinated progression highlights a tightly regulated transition from stress perception to defense execution during infection.

Key regulatory components, including GSTs, COMT, SDRA, and *MLP-like protein 28*, were identified as central nodes potentially linking transcriptional reprogramming with metabolic adjustment and structural defense responses. These regulators may act as key coordination nodes linking transcriptional reprogramming with metabolic and structural defense responses during infection.

Compared with previously reported responses to *Leptosphaeria maculans*, the defense response to *L. biglobosa* appears more gradually activated and temporally phased, suggesting pathogen-specific modulation of host immune timing and intensity.

Collectively, this multi-omics study provides a mechanistic framework for blackleg resistance in *B. napus* and offers candidate genes and pathways for future functional validation and molecular breeding strategies.

## Data Availability

The RNA-seq datasets generated in this study have been deposited in the NCBI Sequence Read Archive (SRA) under accession number PRJNA1475802. The proteomics datasets have been deposited in the iProX database under accession number PXD079373. All data supporting the findings of this study are available within the article and its supplementary materials, and further inquiries can be directed to the corresponding author.
