# Supplementary material for: Integrative transcriptomic and proteomic analysis reveals the regulatory mechanisms underlying oilseed rape resistance to Leptosphaeria biglobosa
Source: Front Plant Sci. 2026 Jul 15;17:1877778. doi: 10.3389/fpls.2026.1877778 (PMC13416561; doi:10.3389/fpls.2026.1877778)
Supplement: Supplementary file 1 [file Table1.docx]

Supplementary Material

# Supplementary Data

Abbreviations

The following abbreviations are used in this manuscript:

| **DEGs** | Differentially Expressed Genes |
| --- | --- |
| **DEPs** | Differentially Expressed Proteins |
| **GO** | Gene Ontology |
| **KEGG** | Kyoto Encyclopedia of Genes and Genomes |
| **MAPK** | Mitogen-Activated Protein Kinase |
| **RNA-seq** | RNA Sequencing |
| **qRT-PCR** | Quantitative Real-Time Polymerase Chain Reaction |
| **POD** | Peroxidase |
| **PAL** | Phenylalanine Ammonia-Lyase |
| **PPO** | Polyphenol Oxidase |
| **MDA** | Malondialdehyde |
| **SOD** | Superoxide Dismutase |
| **GST** | Glutathione S-Transferase |
| **COMT** | Caffeoyl-CoA O-Methyltransferase |
| **MLP** | Major Latex Protein |
| **SDR** | Short-chain Dehydrogenase/Reductase |
| **WGCNA** | Weighted Gene Co-expression Network Analysis |
| **PAMP** | Pathogen-Associated Molecular Pattern |
| **PTI** | PAMP-Triggered Immunity |
| **ETI** | Effector-Triggered Immunity |
| **TMT** | Tandem Mass Tag |
| **CV** | Coefficient of Variation |
| **FPKM** | Fragments Per Kilobase of transcript per Million mapped reads |
| **ROS** | Reactive Oxygen Species |
| **HXK** | Hexokinase |
| **C4H** | Cinnamic Acid 4-Hydroxylase |
| **PDA** | Potato Dextrose Agar |
| **STEM** | Short Time-series Expression Miner |
| **iTRAQ** | Isobaric Tags for Relative and Absolute Quantitation |
| **hpi** | hours post-inoculation |
| **CYP79B2** | Cytochrome P450 79B2 |
| **SOT17** | Sulfotransferase 17 |
| **WRKY** | WRKY Transcription Factor |
| **MYB** | MYB Transcription Factor |
| **NAC** | NAC Transcription Factor |

# Supplementary Figures and Tables

.

| 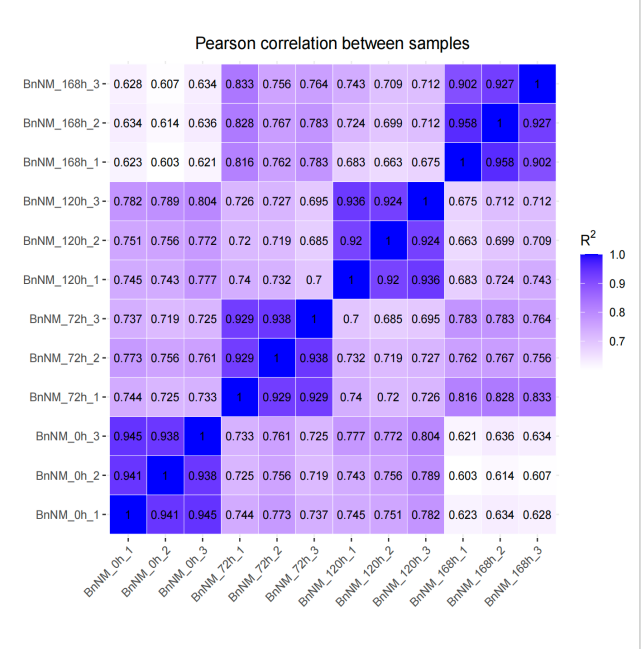 |
| --- |

Supplementary Figure 1.. Sample correlation heatmap

| 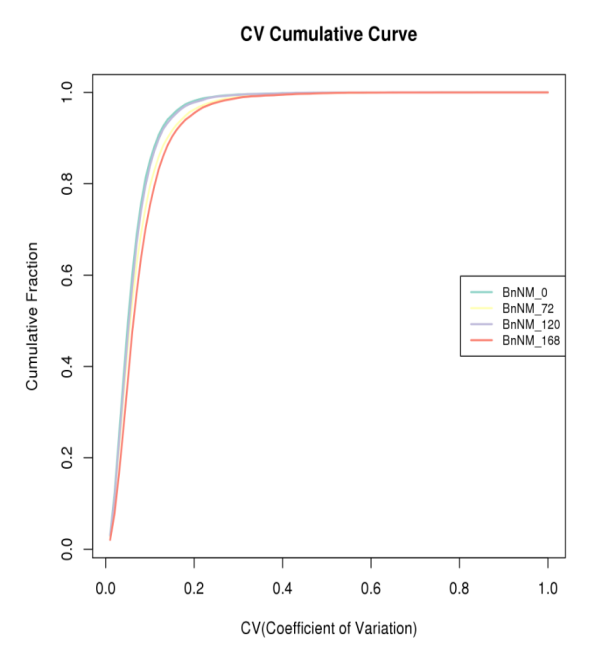 |
| --- |

Supplementary Figure 2.. Repeatability CV analysis

| 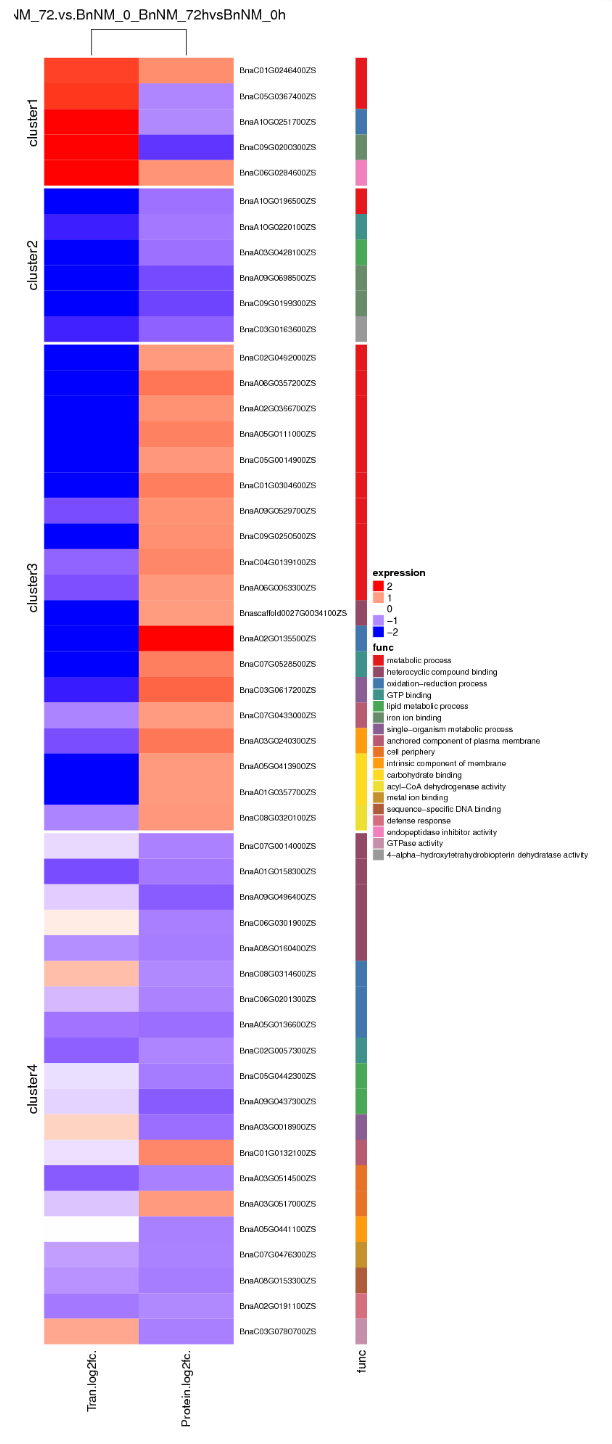 | 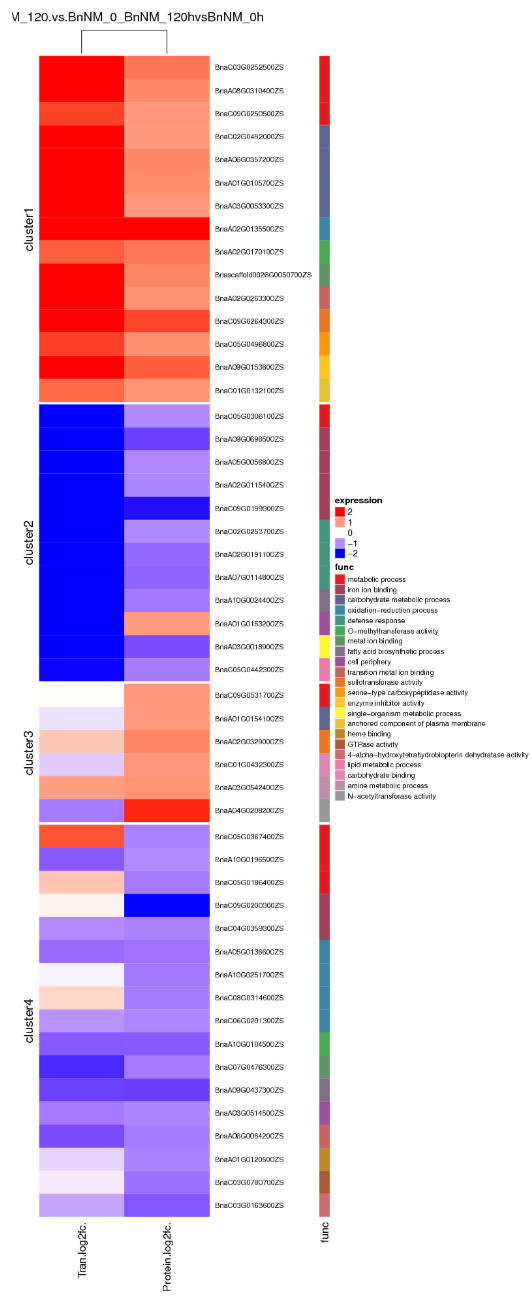 |
| --- | --- |
| (A) | (B) |
| 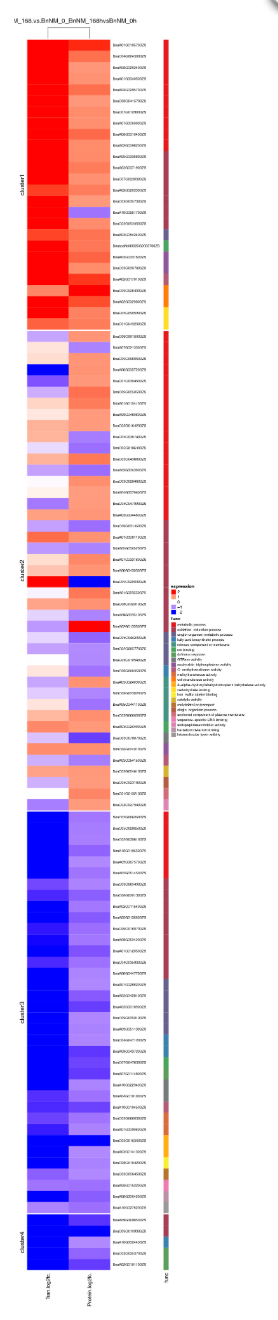 | |
| (C) | |

Supplementary Figure 3.. Correlation analysis of transcriptome and proteome expression levels

Note: Each row in the figure represents a protein. The two columns correspond to the fold change in proteomics data and the fold change in transcriptomics data, respectively. The red sections indicate upregulated proteins, while the blue sections indicate downregulated proteins.

Supplementary Table 1.Candidate defense-associated proteins identified by quantitative proteomic analysis

| Gene ID | Protein Name | Functional Category | 72 h FC | 72 h P value | 120 h FC | 120 h P value | 168 h FC | 168 h P value | Temporal Pattern | Putative Defense Function |
| --- | --- | --- | --- | --- | --- | --- | --- | --- | --- | --- |
| BnaC09G0011200ZS | Glutathione S-transferase F3 (GSTF3) | ROS detoxification | 1.34 | 0.024 | 1.86 | 0.006 | 2.15 | 0.002 | Up–Up–Up | ROS detoxification and redox homeostasis |
| BnaC02G0283300ZS | Glutathione S-transferase U10 (GSTU10) | ROS detoxification | — | — | 1.58 | 0.011 | 1.94 | 0.004 | NC–Up–Up | Antioxidant defense response |
| BnaA02G0170100ZS | Caffeoyl-CoA O-methyltransferase (COMT) | Phenylpropanoid metabolism | 1.12 | 0.087 | 1.42 | 0.038 | 1.79 | 0.009 | Up–Up–Up | Lignin biosynthesis and cell wall strengthening |
| BnaA08G0125600ZS | Short-chain dehydrogenase/reductase (SDRA) | Defense regulation | 1.27 | 0.041 | 1.56 | 0.013 | 1.68 | 0.005 | Up–Up–Up | Redox regulation and lipid metabolism |
| BnaA02G0191100ZS | MLP-like protein 28 | Defense regulation | 0.81 | 0.034 | 0.69 | 0.012 | 0.61 | 0.003 | Down–Down–Down | Defense-associated signaling regulation |
| BnaC02G0253700ZS | MLP-like protein 28 | Defense regulation | — | — | 0.73 | 0.021 | 0.65 | 0.006 | NC–Down–Down | Defense-associated signaling regulation |
| BnaA01G0105700ZS | Chitotriosidase-1 | Antifungal defense | 1.41 | 0.018 | 1.53 | 0.009 | 1.72 | 0.003 | Up–Up–Up | Fungal cell wall degradation |
| BnaA02G0263300ZS | Primary amine oxidase | ROS metabolism | — | — | 1.49 | 0.017 | 1.66 | 0.006 | NC–Up–Up | ROS production and defense signaling |

Note: Candidate defense-associated proteins were selected from significantly differentially expressed proteins (DEPs) identified by TMT-based proteomics (FC ≥ 1.20 or ≤ 0.83, raw P value < 0.05). These proteins were further prioritized based on their functional relevance to ROS detoxification, MAPK signaling, phenylpropanoid biosynthesis, cell wall modification, and antifungal defense. The selected proteins represent key components of stage-specific immune responses during L. biglobosa infection in Brassica napus.

Supplementary Table 2.Candidate defense-associated genes identified by transcriptomic analysis

| Gene ID | Gene Symbol | Functional Category | 72 h log2FC | 72 h padj | 120 h log2FC | 120 h padj | 168 h log2FC | 168 h padj |
| --- | --- | --- | --- | --- | --- | --- | --- | --- |
| BnaC04G0504700ZS | GST | ROS detoxification | 5.318 | 2.57E−160 | 2.363 | 1.86E−20 | 4.177 | 3.71E−110 |
| BnaC04G0415600ZS | DJ-1 homolog | ROS detoxification / stress response | 3.666 | 3.24E−58 | 1.542 | 6.36E−05 | 3.729 | 2.25E−35 |
| BnaA02G0024800ZS | WRKY TF | MAPK / immune signaling | 3.535 | 3.87E−27 | 2.301 | 4.16E−12 | 2.563 | 2.54E−18 |
| BnaC03G0325300ZS | MEKK1 | MAPK signaling | −1.211 | 2.34E−12 | 0.078 | 0.839 | −2.139 | 3.87E−17 |
| BnaA04G0237600ZS | PAL1 | Phenylpropanoid biosynthesis | 2.918 | 7.35E−04 | 0.074 | 0.971 | 1.740 | 0.156 |
| BnaA02G0403000ZS | 4CL | Phenylpropanoid biosynthesis | 1.032 | 1.64E−05 | 1.143 | 2.91E−06 | 0.405 | 0.219 |
| BnaA02G0322800ZS | CCR1 | Phenylpropanoid biosynthesis | −1.743 | 3.49E−13 | −1.200 | 3.55E−07 | −2.252 | 1.12E−16 |
| BnaA08G0190200ZS | CAD8 | Phenylpropanoid biosynthesis | 5.753 | 7.16E−07 | −2.803 | 0.220 | 2.336 | 2.00E−02 |
| BnaC02G0144400ZS | XTH22 | Cell wall remodeling | −2.944 | 6.48E−18 | −9.479 | 2.64E−35 | −5.585 | 1.53E−37 |
| BnaA04G0187100ZS | Expansin-A6 | Cell wall remodeling | −4.571 | 7.49E−63 | −5.898 | 3.14E−57 | −7.608 | 4.09E−57 |
| BnaA02G0399800ZS | PMEI | Cell wall remodeling | 3.378 | 2.01E−46 | −0.534 | 0.201 | −0.257 | 0.529 |
| BnaC03G0815500ZS | Cellulose synthase-like | Cell wall remodeling | −1.314 | 2.57E−07 | 0.676 | 1.45E−02 | −0.035 | 0.922 |
| BnaA09G0203600ZS | Subtilisin-like protease | Antifungal defense | 1.786 | 3.20E−17 | 1.467 | 8.06E−08 | −0.467 | 0.127 |
| novel.3459 | PR5-like protein | Antifungal defense | −7.548 | 1.96E−19 | −9.340 | 1.18E−12 | −10.295 | 1.21E−15 |
| novel.1558 | MLP28 homolog | Candidate regulator | −3.063 | 2.97E−17 | −0.699 | 0.110 | −0.657 | 0.092 |

Note：Candidate defense-associated genes were selected from significantly differentially expressed genes (DEGs) identified by RNA-seq analysis (|log2FC| ≥ 1, padj ≤ 0.05). Genes were prioritized based on their involvement in reactive oxygen species detoxification, MAPK signaling pathway, phenylpropanoid biosynthesis, cell wall remodeling, and defense-related regulatory processes. These genes represent key transcriptional regulators underlying temporal immune responses during L. biglobosa infection.
